# Crystal structure of poly[(2,2′-bi­pyridine-κ^2^ *N*,*N*′)tetra-μ_2_-cyanido-κ^4^ *C*:*N*;κ^4^ *N*:*C*-manganese(II)disilver(I)]

**DOI:** 10.1107/S205698901501676X

**Published:** 2015-09-12

**Authors:** Chatphorn Theppitak, Kittipong Chainok

**Affiliations:** a Department of Chemistry, Faculty of Science, Naresuan University, Muang, Phitsanulok, 65000, Thailand; b Department of Physics, Faculty of Science and Technology, Thammasat University, Khlong Luang, Pathum Thani, 12120, Thailand

**Keywords:** crystal structure, di­cyano­argentate(I), manganese(II), triple inter­penetration

## Abstract

The title compound, [Ag_2_Mn(CN)_4_(C_10_H_8_N_2_)]_*n*_ or Mn(bipy){Ag(CN)_2_}_2_ (bipy = 2,2′-bi­pyridine) is isostructural with Cd(bipy){Au(CN)_2_}_2_ [Guo *et al.* (2009 ▸). *CrystEngComm*, **11**, 61–66]. The Mn^II^ atom has crystallographically imposed twofold symmetry and a distorted octa­hedral coordination sphere consisting of six N atoms from one bi­pyridine ligand and four di­cyano­argentate(I) anions, [Ag(CN)_2_]^−^, while the Ag^I^ atom of the complex anion displays the expected linear geometry. Each [Ag(CN)_2_]^−^ unit connects to two neighbouring [Mn(bipy)]^2+^ cations to give an threefold inter­penetrating quartz-like three-dimensional framework. No directional inter­actions beyond van der Waals contacts are observed.

## Related literature   

For related crystal structures, see: Soma *et al.* (1994 ▸); Guo *et al.* (2009 ▸). For the use of [Ag(CN)_2_]^−^ as a building block for the construction of cyanide-bridged silver(I)–iron(II) spin-crossover coordination polymers, see: Shorrock *et al.* (2002 ▸); Galet *et al.* (2003 ▸); Niel *et al.* (2003 ▸); Muñoz *et al.* (2007 ▸). 
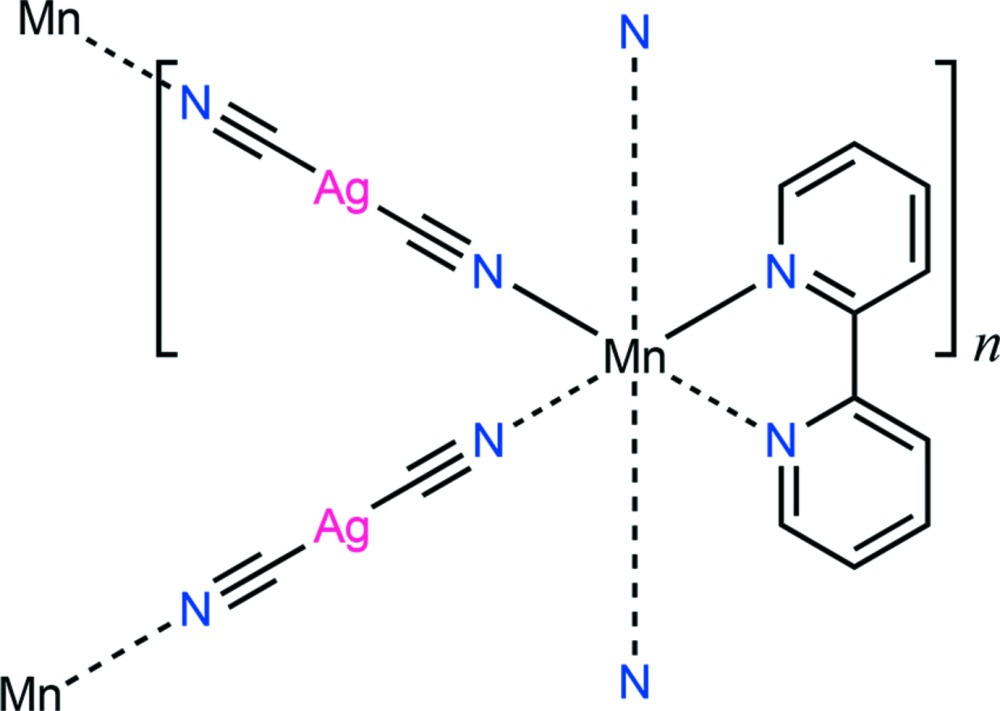


## Experimental   

### Crystal data  

[Ag_2_Mn(CN)_4_(C_10_H_8_N_2_)]
*M* *_r_* = 530.94
Trigonal, 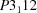

*a* = 8.7215 (3) Å
*c* = 20.9874 (9) Å
*V* = 1382.52 (13) Å^3^
*Z* = 3
Mo *K*α radiation
μ = 2.78 mm^−1^
*T* = 296 K
0.36 × 0.22 × 0.22 mm

### Data collection  

Bruker D8 QUEST CMOS diffractometer
Absorption correction: multi-scan (*SADABS*; Bruker,2014 ▸) *T*_min_ = 0.484, *T*_max_ = 0.542
25845 measured reflections
1874 independent reflections
1856 reflections with *I* > 2σ(*I*)
*R*_int_ = 0.024

### Refinement  

*R*[*F* ^2^ > 2σ(*F* ^2^)] = 0.015
*wR*(*F* ^2^) = 0.038
*S* = 1.07
1874 reflections
105 parameters
H-atom parameters constrained
Δρ_max_ = 0.15 e Å^−3^
Δρ_min_ = −0.28 e Å^−3^
Absolute structure: Flack *x* determined using 824 quotients [(*I* ^+^)−(*I* ^−^)]/[(*I* ^+^)+(*I* ^−^)] (Parsons *et al.*, 2013 ▸)
Absolute structure parameter: 0.037 (6)

### 

Data collection: *APEX2* (Bruker, 2014 ▸); cell refinement: *SAINT* (Bruker, 2014 ▸); data reduction: *SAINT*; program(s) used to solve structure: *SHELXT* (Sheldrick, 2015*a* ▸); program(s) used to refine structure: *SHELXL2014* (Sheldrick, 2015*b* ▸); molecular graphics: *OLEX2* (Dolomanov *et al.*, 2009 ▸); software used to prepare material for publication: *publCIF* (Westrip, 2010 ▸) and *enCIFer* (Allen *et al.*, 2004 ▸).

## Supplementary Material

Crystal structure: contains datablock(s) I. DOI: 10.1107/S205698901501676X/vn2098sup1.cif

Structure factors: contains datablock(s) I. DOI: 10.1107/S205698901501676X/vn2098Isup2.hkl

Supporting information file. DOI: 10.1107/S205698901501676X/vn2098Isup3.cdx

**Fig. 1.**
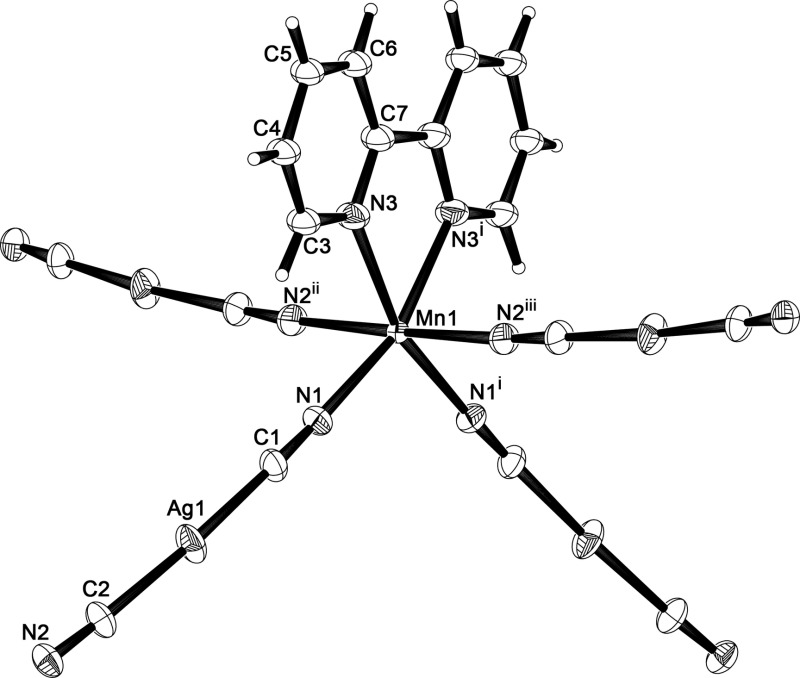
Displacement ellipsoid plot at the 35% probability level of the immediate coordination geometry about the manganese(II) centre in the title compound. The asymmetric unit is labelled. [Symmetry codes: (i) x, -1 + x – y, 2 – z; (ii) 1 – y, x – y, 1/3 + z; (iii) 1 – y, –x, 5/3 – z].

**Fig. 2.**
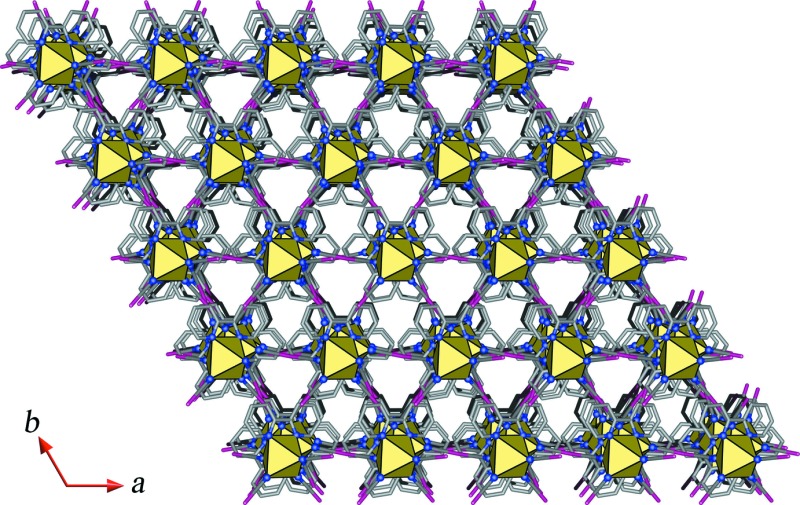
Crystal packing of the title compound viewed along c axis.

Click here for additional data file.x x y z y x y z y z . DOI: 10.1107/S205698901501676X/vn2098fig1.tif
Displacement ellipsoid plot at the 35% probability level of the immediate coordination geometry about the manganese(II) centre in the title compound. The asymmetric unit is labelled. [Symmetry codes: (i) *x*, −1 + *x* – *y*, 2 – *z*; (ii) 1 – *y*, *x* – *y*, 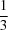 + *z*; (iii) 1 – *y*, –x, 5/3 – *z*].

c . DOI: 10.1107/S205698901501676X/vn2098fig2.tif
Crystal packing of the title compound viewed along *c* axis.

CCDC reference: 1422937

Additional supporting information:  crystallographic information; 3D view; checkCIF report

## supplementary crystallographic information

### S1. Synthesis and crystallization

Mn(NO_3_)_2_·6H_2_O (62 mg, 0.5 mmol) and 2,2'-bi­pyridine (162 mg, 0.5 mmol) were dissolved in 4 ml of a mixture H_2_O/CH_3_OH (1:1) to form a bright yellow solution and this was pipetted into one side of the H-tube. K[Ag(CN)_2_] (250 mg, 2 mmol) was dissolved in 4 mL of a mixture H_2_O/CH_3_OH to give a colorless solution and this was pipetted into the other side arm of the H-tube. The H-tube was then carefully filled with a mixture H_2_O/CH_3_OH. Upon slow diffusion for 3 days, pale-yellow block shaped single crystals of the title compound were formed in the manganese(II)-containing side of the H-tube. Yield: 49 mg, 89% based on manganese source.

### S2. Refinement

The C–bound hydrogen atoms were placed in geometrically idealized positions based on chemical coordinations and constrained to ride on their parent atom positions with a C—H distances of 0.93 Å and with *U*_iso_(H) = 1.2*U*_eq_(C) for the aromatic H atoms.

### Crystal data

**Table:**

[Ag_2_Mn(CN)_4_(C_10_H_8_N_2_)]	*D*_x_ = 1.913 Mg m^−^^3^
*M**_r_* = 530.94	Mo *K*α radiation, λ = 0.71073 Å
Trigonal, *P*3_1_12	Cell parameters from 720 reflections
*a* = 8.7215 (3) Å	θ = 3.3–26.3°
*c* = 20.9874 (9) Å	µ = 2.78 mm^−^^1^
*V* = 1382.52 (13) Å^3^	*T* = 296 K
*Z* = 3	Block, light yellow
*F*(000) = 759	0.36 × 0.22 × 0.22 mm

### Data collection

**Table:**

Bruker D8 QUEST CMOS diffractometer	1874 independent reflections
Radiation source: microfocus sealed x-ray tube, Incoatec Iµus	1856 reflections with *I* > 2σ(*I*)
GraphiteDouble Bounce Multilayer Mirror monochromator	*R*_int_ = 0.024
Detector resolution: 10.5 pixels mm^-1^	θ_max_ = 26.3°, θ_min_ = 3.3°
ω and φ scans	*h* = −10→10
Absorption correction: multi-scan (*SADABS*; Bruker,2014)	*k* = −10→10
*T*_min_ = 0.484, *T*_max_ = 0.542	*l* = −26→26
25845 measured reflections	

### Refinement

**Table:**

Refinement on *F*^2^	Hydrogen site location: inferred from neighbouring sites
Least-squares matrix: full	H-atom parameters constrained
*R*[*F*^2^ > 2σ(*F*^2^)] = 0.015	*w* = 1/[σ^2^(*F*_o_^2^) + (0.0231*P*)^2^ + 0.1949*P*] where *P* = (*F*_o_^2^ + 2*F*_c_^2^)/3
*wR*(*F*^2^) = 0.038	(Δ/σ)_max_ = 0.001
*S* = 1.07	Δρ_max_ = 0.15 e Å^−^^3^
1874 reflections	Δρ_min_ = −0.28 e Å^−^^3^
105 parameters	Absolute structure: Flack *x* determined using 824 quotients [(*I*^+^)-(*I*^-^)]/[(*I*^+^)+(*I*^-^)] (Parsons *et al.*, 2013)
0 restraints	Absolute structure parameter: 0.037 (6)
Primary atom site location: structure-invariant direct methods	

### Special details

**Table:**

Geometry. All e.s.d.'s (except the e.s.d. in the dihedral angle between two l.s. planes) are estimated using the full covariance matrix. The cell e.s.d.'s are taken into account individually in the estimation of e.s.d.'s in distances, angles and torsion angles; correlations between e.s.d.'s in cell parameters are only used when they are defined by crystal symmetry. An approximate (isotropic) treatment of cell e.s.d.'s is used for estimating e.s.d.'s involving l.s. planes.

### Fractional atomic coordinates and isotropic or equivalent isotropic displacement parameters (Å^2^)

**Table:**

	*x*	*y*	*z*	*U*_iso_*/*U*_eq_	
Ag1	0.46725 (4)	0.05832 (4)	0.84789 (2)	0.07435 (11)	
Mn1	0.89941 (6)	−0.05030 (3)	1.0000	0.03424 (11)	
N2	0.2249 (4)	0.0953 (4)	0.74313 (13)	0.0600 (6)	
N3	1.1424 (3)	0.1907 (3)	0.96099 (11)	0.0511 (5)	
N1	0.7178 (3)	−0.0053 (4)	0.94131 (11)	0.0547 (6)	
C7	1.3017 (3)	0.2186 (3)	0.97859 (12)	0.0438 (5)	
C4	1.2821 (5)	0.4635 (6)	0.9044 (3)	0.0981 (17)	
H4	1.2710	0.5468	0.8803	0.118*	
C2	0.3101 (5)	0.0826 (5)	0.78142 (16)	0.0638 (8)	
C1	0.6284 (4)	0.0198 (5)	0.90861 (14)	0.0612 (7)	
C6	1.4560 (4)	0.3673 (4)	0.95830 (15)	0.0599 (7)	
H6	1.5658	0.3828	0.9698	0.072*	
C3	1.1344 (5)	0.3099 (6)	0.9239 (2)	0.0848 (13)	
H3	1.0238	0.2887	0.9106	0.102*	
C5	1.4453 (4)	0.4911 (5)	0.92118 (18)	0.0807 (11)	
H5	1.5474	0.5920	0.9077	0.097*	

### Atomic displacement parameters (Å^2^)

**Table:**

	*U*^11^	*U*^22^	*U*^33^	*U*^12^	*U*^13^	*U*^23^
Ag1	0.0916 (2)	0.1011 (2)	0.05771 (15)	0.06867 (18)	−0.02819 (13)	−0.00888 (13)
Mn1	0.0307 (2)	0.03718 (18)	0.0327 (2)	0.01535 (11)	0.000	−0.00120 (16)
N2	0.0675 (15)	0.0575 (14)	0.0588 (14)	0.0342 (13)	−0.0179 (12)	0.0000 (12)
N3	0.0375 (11)	0.0535 (13)	0.0546 (12)	0.0170 (9)	0.0022 (9)	0.0141 (10)
N1	0.0530 (13)	0.0719 (16)	0.0470 (12)	0.0371 (12)	−0.0084 (10)	−0.0012 (12)
C7	0.0365 (11)	0.0497 (13)	0.0359 (11)	0.0146 (10)	0.0005 (9)	−0.0054 (9)
C4	0.063 (2)	0.080 (3)	0.124 (4)	0.0157 (19)	0.000 (2)	0.056 (3)
C2	0.0752 (19)	0.0685 (19)	0.0578 (17)	0.0435 (17)	−0.0227 (14)	−0.0033 (14)
C1	0.0654 (18)	0.081 (2)	0.0514 (15)	0.0473 (18)	−0.0097 (13)	−0.0030 (14)
C6	0.0363 (13)	0.0621 (17)	0.0594 (16)	0.0082 (12)	−0.0054 (11)	0.0016 (13)
C3	0.0467 (17)	0.082 (2)	0.109 (3)	0.0199 (17)	−0.0021 (18)	0.050 (2)
C5	0.0511 (17)	0.064 (2)	0.088 (2)	−0.0001 (14)	0.0002 (16)	0.0260 (18)

### Geometric parameters (Å, º)

**Table:**

Ag1—C2	2.039 (3)	N3—C3	1.328 (4)
Ag1—C1	2.046 (3)	N1—C1	1.139 (4)
Mn1—N2^i^	2.229 (3)	C7—C7^iii^	1.485 (5)
Mn1—N2^ii^	2.229 (3)	C7—C6	1.388 (4)
Mn1—N3^iii^	2.264 (2)	C4—H4	0.9300
Mn1—N3	2.264 (2)	C4—C3	1.377 (5)
Mn1—N1^iii^	2.192 (2)	C4—C5	1.365 (6)
Mn1—N1	2.192 (2)	C6—H6	0.9300
N2—Mn1^iv^	2.229 (3)	C6—C5	1.372 (5)
N2—C2	1.137 (4)	C3—H3	0.9300
N3—C7	1.337 (3)	C5—H5	0.9300
			
C2—Ag1—C1	174.70 (14)	C3—N3—C7	118.4 (2)
N2^i^—Mn1—N2^ii^	177.94 (15)	C1—N1—Mn1	177.0 (3)
N2^ii^—Mn1—N3	85.78 (10)	N3—C7—C7^iii^	115.84 (15)
N2^ii^—Mn1—N3^iii^	92.55 (10)	N3—C7—C6	121.3 (3)
N2^i^—Mn1—N3^iii^	85.78 (10)	C6—C7—C7^iii^	122.90 (17)
N2^i^—Mn1—N3	92.55 (10)	C3—C4—H4	120.6
N3^iii^—Mn1—N3	71.65 (12)	C5—C4—H4	120.6
N1^iii^—Mn1—N2^i^	92.34 (11)	C5—C4—C3	118.7 (4)
N1—Mn1—N2^i^	88.95 (10)	N2—C2—Ag1	178.2 (3)
N1—Mn1—N2^ii^	92.34 (10)	N1—C1—Ag1	178.1 (3)
N1^iii^—Mn1—N2^ii^	88.94 (10)	C7—C6—H6	120.2
N1—Mn1—N3	93.18 (10)	C5—C6—C7	119.6 (3)
N1^iii^—Mn1—N3	163.66 (9)	C5—C6—H6	120.2
N1^iii^—Mn1—N3^iii^	93.18 (10)	N3—C3—C4	123.1 (3)
N1—Mn1—N3^iii^	163.66 (9)	N3—C3—H3	118.5
N1^iii^—Mn1—N1	102.49 (15)	C4—C3—H3	118.5
C2—N2—Mn1^iv^	176.1 (3)	C4—C5—C6	118.9 (3)
C7—N3—Mn1	118.32 (18)	C4—C5—H5	120.6
C3—N3—Mn1	123.2 (2)	C6—C5—H5	120.6
			
Mn1—N3—C7—C7^iii^	1.5 (4)	C7—C6—C5—C4	−0.8 (7)
Mn1—N3—C7—C6	−177.6 (2)	C3—N3—C7—C7^iii^	178.4 (4)
Mn1—N3—C3—C4	174.7 (5)	C3—N3—C7—C6	−0.7 (5)
N3—C7—C6—C5	2.2 (5)	C3—C4—C5—C6	−1.8 (9)
C7—N3—C3—C4	−2.0 (7)	C5—C4—C3—N3	3.3 (10)
C7^iii^—C7—C6—C5	−176.9 (4)		

Symmetry codes: (i) −*y*+1, −*x*, −*z*+5/3; (ii) −*y*+1, *x*−*y*, *z*+1/3; (iii) *x*, *x*−*y*−1, −*z*+2; (iv) −*x*+*y*+1, −*x*+1, *z*−1/3.

## References

[bb1] Allen, F. H., Johnson, O., Shields, G. P., Smith, B. R. & Towler, M. (2004). *J. Appl. Cryst.* **37**, 335–338.

[bb2] Bruker (2014). *APEX2*, *SADABS* and *SAINT*. Bruker AXS Inc., Madison, Wisconsin, USA.

[bb3] Dolomanov, O. V., Bourhis, L. J., Gildea, R. J., Howard, J. A. K. & Puschmann, H. (2009). *J. Appl. Cryst.* **42**, 339–341.

[bb4] Galet, A., Niel, V., Muñoz, M. C. & Real, J. A. (2003). *J. Am. Chem. Soc.* **125**, 14224–14225. 10.1021/ja0377347 14624540

[bb5] Guo, Y., Liu, Z.-Q., Zhao, B., Feng, Y.-H., Xu, G.-F., Yan, S.-P., Cheng, P., Wang, Q.-L. & Liao, D.-Z. (2009). *CrystEngComm*, **11**, 61–66.

[bb6] Muñoz, M. C., Gaspar, A. B., Galet, A. & Real, J. A. (2007). *Inorg. Chem.* **46**, 8182–8192. 10.1021/ic700607x 17764171

[bb7] Niel, V., Thompson, A. L., Muñoz, M. C., Galet, A., Goeta, A. E. & Real, J. A. (2003). *Angew. Chem. Int. Ed.* **42**, 3760–3763. 10.1002/anie.200351853 12923837

[bb8] Parsons, S., Flack, H. D. & Wagner, T. (2013). *Acta Cryst.* B**69**, 249–259. 10.1107/S2052519213010014 PMC3661305 23719469

[bb9] Sheldrick, G. M. (2015*a*). *Acta Cryst.* A**71**, 3–8.

[bb10] Sheldrick, G. M. (2015*b*). *Acta Cryst.* C**71**, 3–8.

[bb11] Shorrock, C. J., Xue, B.-Y., Kim, P. B., Batchelor, R. J., Patrick, B. O. & Leznoff, D. B. (2002). *Inorg. Chem.* **41**, 6743–6753. 10.1021/ic025850p 12470070

[bb12] Soma, T., Yuge, H. & Iwamoto, T. (1994). *Angew. Chem. Int. Ed. Engl.* **33**, 1665–1666.

[bb13] Westrip, S. P. (2010). *J. Appl. Cryst.* **43**, 920–925.

