# Supplementary figures and images for: Crystal structure of poly[(2,2′-bi­pyridine-κ2 N,N′)tetra-μ2-cyanido-κ4 C:N;κ4 N:C-manganese(II)disilver(I)]
Source: Acta Crystallogr E Crystallogr Commun. 2015 Sep 12;71(Pt 10):m179–80. doi: 10.1107/S205698901501676X (PMC4647358; doi:10.1107/S205698901501676X)

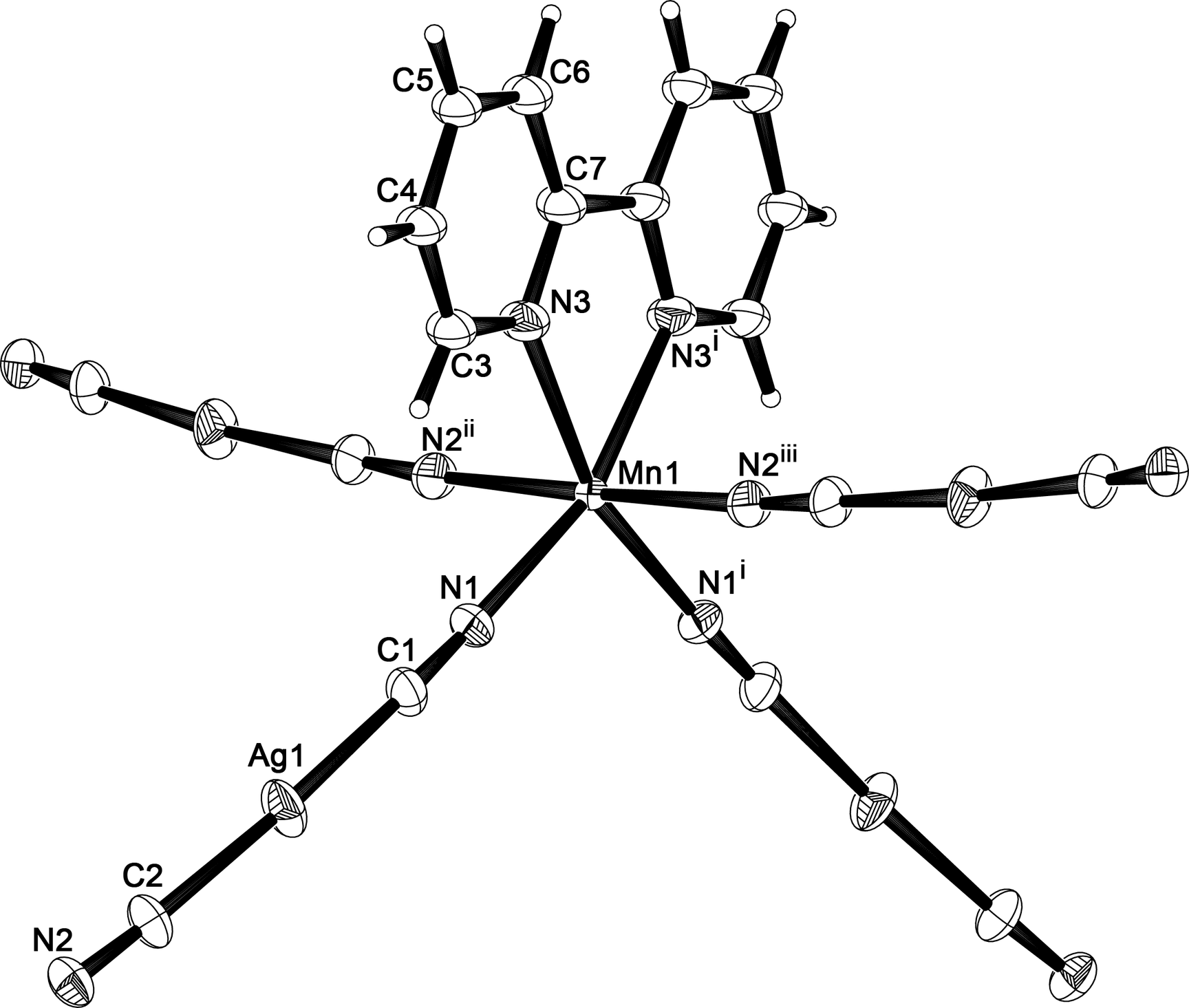

Supplement: Supplementary file 4 [file e-71-0m179-fig1.tif]

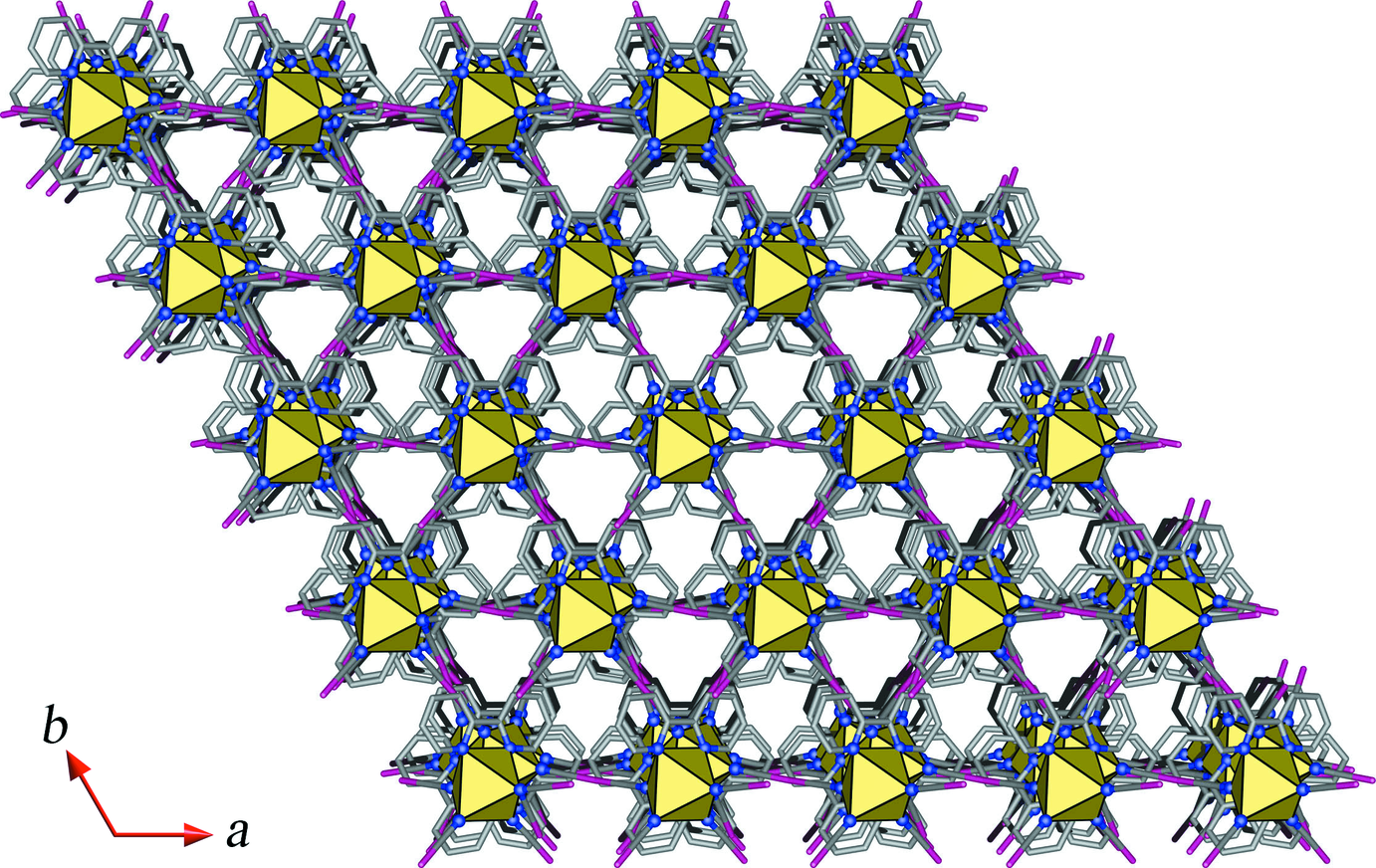

Supplement: Supplementary file 5 [file e-71-0m179-fig2.tif]
